# Geospatial blockchain: promises, challenges, and scenarios in health and healthcare

**DOI:** 10.1186/s12942-018-0144-x

**Published:** 2018-07-05

**Authors:** Maged N. Kamel Boulos, James T. Wilson, Kevin A. Clauson

**Affiliations:** 1 Moray College, University of the Highlands and Islands, Elgin, IV30 1JJ Scotland, UK; 2 Lipscomb University College of Pharmacy and Health Sciences, Nashville, TN 37204-3951 USA

**Keywords:** Blockchain, Geospatial blockchain, Crypto-spatial coordinate system, Cryptography, Distributed ledger technology, Smart contracts, Internet of Things, Smart cities, Clinical trials, Supply chain, Pharmaceuticals, Healthcare

## Abstract

A PubMed query run in June 2018 using the keyword ‘blockchain’ retrieved 40 indexed papers, a reflection of the growing interest in blockchain among the medical and healthcare research and practice communities. Blockchain’s foundations of decentralisation, cryptographic security and immutability make it a strong contender in reshaping the healthcare landscape worldwide. Blockchain solutions are currently being explored for: (1) securing patient and provider identities; (2) managing pharmaceutical and medical device supply chains; (3) clinical research and data monetisation; (4) medical fraud detection; (5) public health surveillance; (6) enabling truly public and open geo-tagged data; (7) powering many Internet of Things-connected autonomous devices, wearables, drones and vehicles, via the distributed peer-to-peer apps they run, to deliver the full vision of smart healthy cities and regions; and (8) blockchain-enabled augmented reality in crisis mapping and recovery scenarios, including mechanisms for validating, crediting and rewarding crowdsourced geo-tagged data, among other emerging use cases. Geospatially-enabled blockchain solutions exist today that use a crypto-spatial coordinate system to add an immutable spatial context that regular blockchains lack. These geospatial blockchains do not just record an entry’s specific time, but also require and validate its associated proof of location, allowing accurate spatiotemporal mapping of physical world events. Blockchain and distributed ledger technology face similar challenges as any other technology threatening to disintermediate legacy processes and commercial interests, namely the challenges of blockchain interoperability, security and privacy, as well as the need to find suitable and sustainable business models of implementation. Nevertheless, we expect blockchain technologies to get increasingly powerful and robust, as they become coupled with artificial intelligence (AI) in various real-word healthcare solutions involving AI-mediated data exchange on blockchains.

## Background

In order to understand the utility and disruptive potential that blockchain technology offers, one must first review the fundamentals of the technology itself. Blockchain is a decentralised, immutable, and cryptographically secure distributed ledger technology (DLT), broadly used to eliminate the need for trust in data transfer, and well known for powering the Bitcoin cryptocurrency [1]. Our goal with this article is to review recent, state-of-the-art blockchain uses in healthcare, particularly uses involving a geospatial component. To achieve this goal, we first need to examine the properties of blockchains more closely to learn why they are vital and what the technology aims to accomplish.

The distribution element of blockchain as a distributed ledger refers to the design of the system on which the blockchain is running (i.e., how many computers are contained in the system) and the number of individuals or organisations that control or own said computers. DLTs are built on consensus utilising algorithms to find agreement among participants [e.g., Proof of Work (PoW), Proof of Stake (PoS)], data replication, and peer-to-peer (P2P) networking. Decentralisation is a subset of distribution concerning ownership and control of the data on the system and decisions about the system itself [2]. As Vitalik Buterin, co-founder of Ethereum, writes: “Blockchains are politically decentralized (no one controls them) and architecturally decentralized (no infrastructural central point of failure) but they are logically centralized (there is one commonly agreed state and the system behaves like a single computer” [2]. Decentralisation allows for resistance to system failure, attacks and manipulation, and participant collusion. Put simply, increasing the number of participants (i.e., computers, nodes) and the number of unique owners across the system decreases the chance of an overall system failure or takeover. If one computer is storing all data and that computer fails or is hacked, the system cannot recover. Decentralisation largely prevents this from occurring.

Cryptography is another major underpinning of blockchain technology responsible for several major functions, including proof of data/asset ownership and data validation. Two forms of cryptography commonly employed with blockchains are one-way hashing functions, such as SHA-256 (Secure Hashing Algorithm), and asymmetric encryption (i.e., two-way function) utilising public and private keys [1, 3]. Each of these tools has a role in securing and proving ownership and preventing non-consensus driven modifications to the ledger. Let us look at an example of each to understand how they work and what exactly they are doing when used for blockchain transactions. It is important to recognise that while initial blockchain transactions were financial in nature and applied exclusively to cryptocurrencies, blockchain transactions can refer to transfer of any digital asset—including data.

In the case of a one-way hashing function (e.g., SHA-256), the hash of data put into the function cannot be used algorithmically to find what the original data were [4]. One example of its utility is if we downloaded a program from the Internet, but not directly from the developer’s website, and we wanted to verify that the program has not been tampered with in any way—malicious or otherwise. In many cases, the software developers will provide hash sums to double-check for this specific purpose. Suppose the hash sum provided is ‘ce28b8951318f4f3a54c7009dc783c13be8db90e074c0bb024635daa91b0bbe7’; if the calculated hash of the downloaded program does not match this, it has been tampered with in some way. Small changes can lead to huge differences in the hash sum, which are easy to identify.

**Fig. 1:**
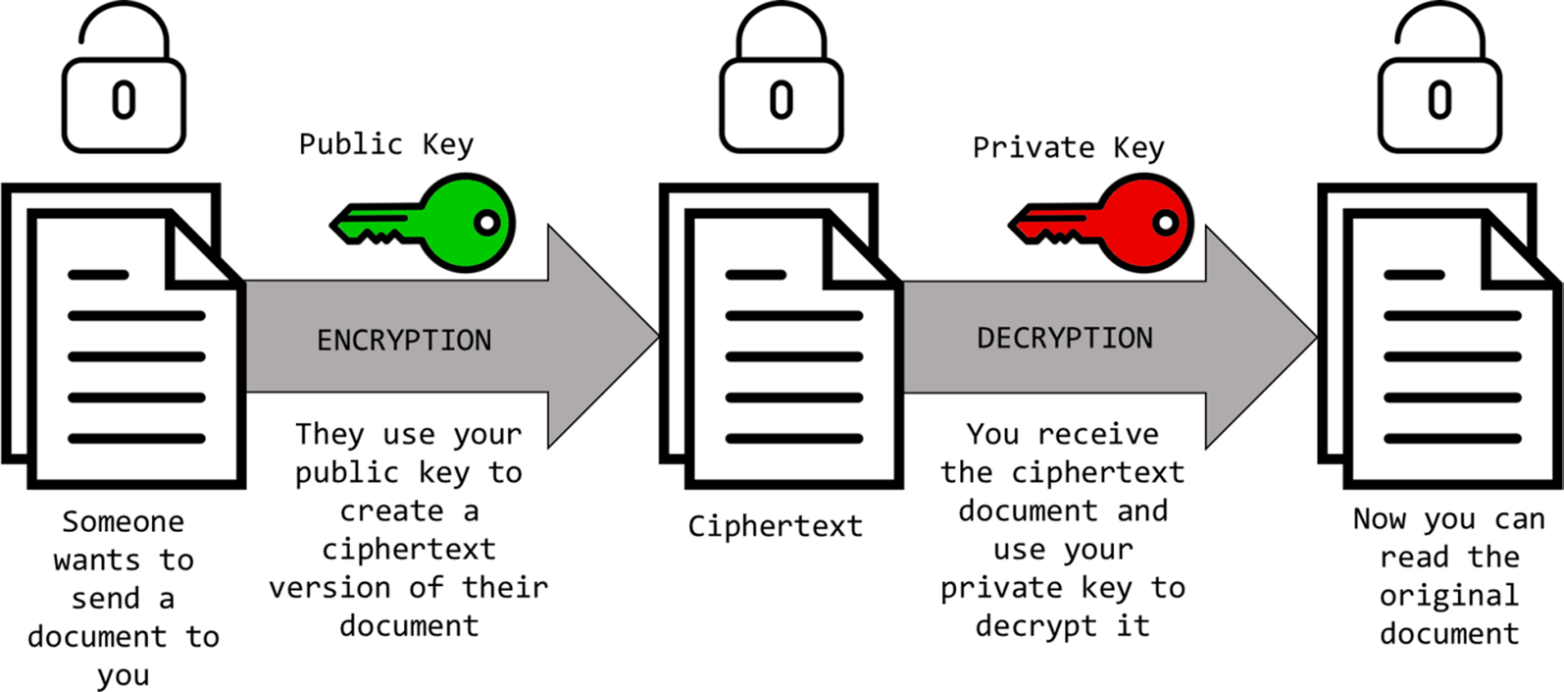
Using shared public key to encrypt document from outside party

**Fig. 2:**
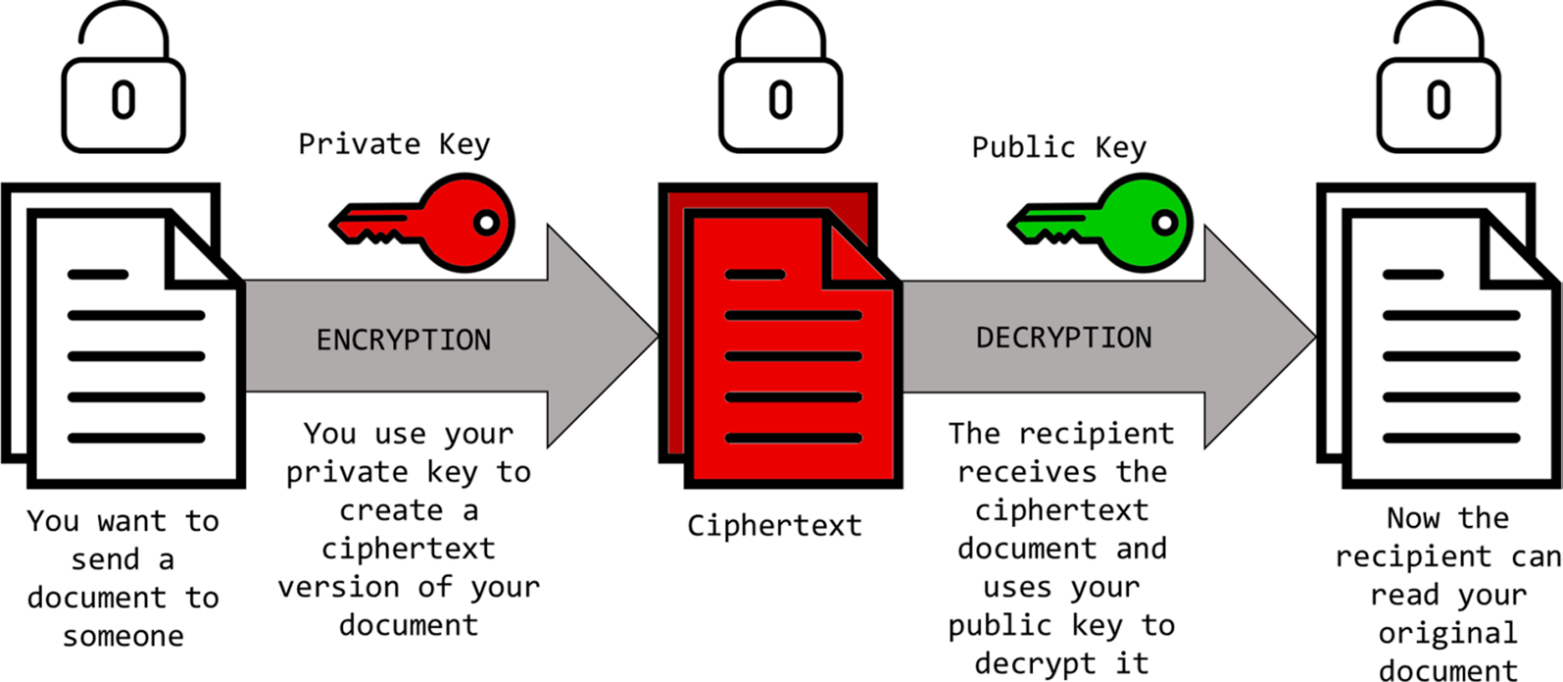
Using private key to encrypt document to be read by outside party with appropriate public key

Asymmetric encryption, known as public key encryption, is a two-way cryptographic function. It will begin with data and encrypt or scramble them using a key pair, rendering them (the data) useless if they ended up in the possession of anyone not in possession of the requisite key. These encrypted data, however, can be decrypted by the receiving party if they possess the correct key. Public key encryption can be used in two basic ways: to encrypt data that only the private key holder can decrypt and use, and to prove that data came from a trusted source by “signing” with a private key. Imagine a document containing sensitive information while examining two use cases for asymmetric encryption [5]. Figure 1 depicts the encryption of a document from an outside party utilising the first party’s public key, whereas Fig. 2 illustrates encryption of a document from the first party using their private key to be read by an outside party in possession of the appropriate public key.

**Fig. 3:**
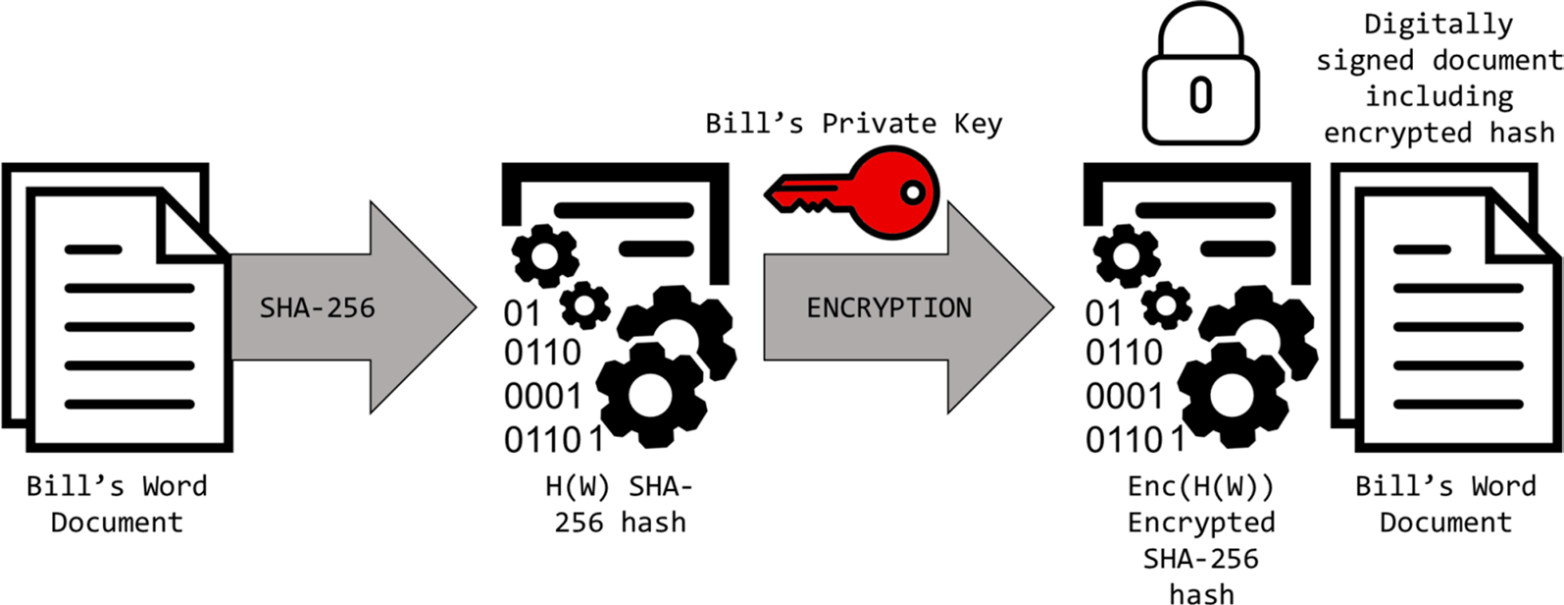
Using asymmetric encryption in addition to hashing to digitally sign a document

**Fig. 4:**
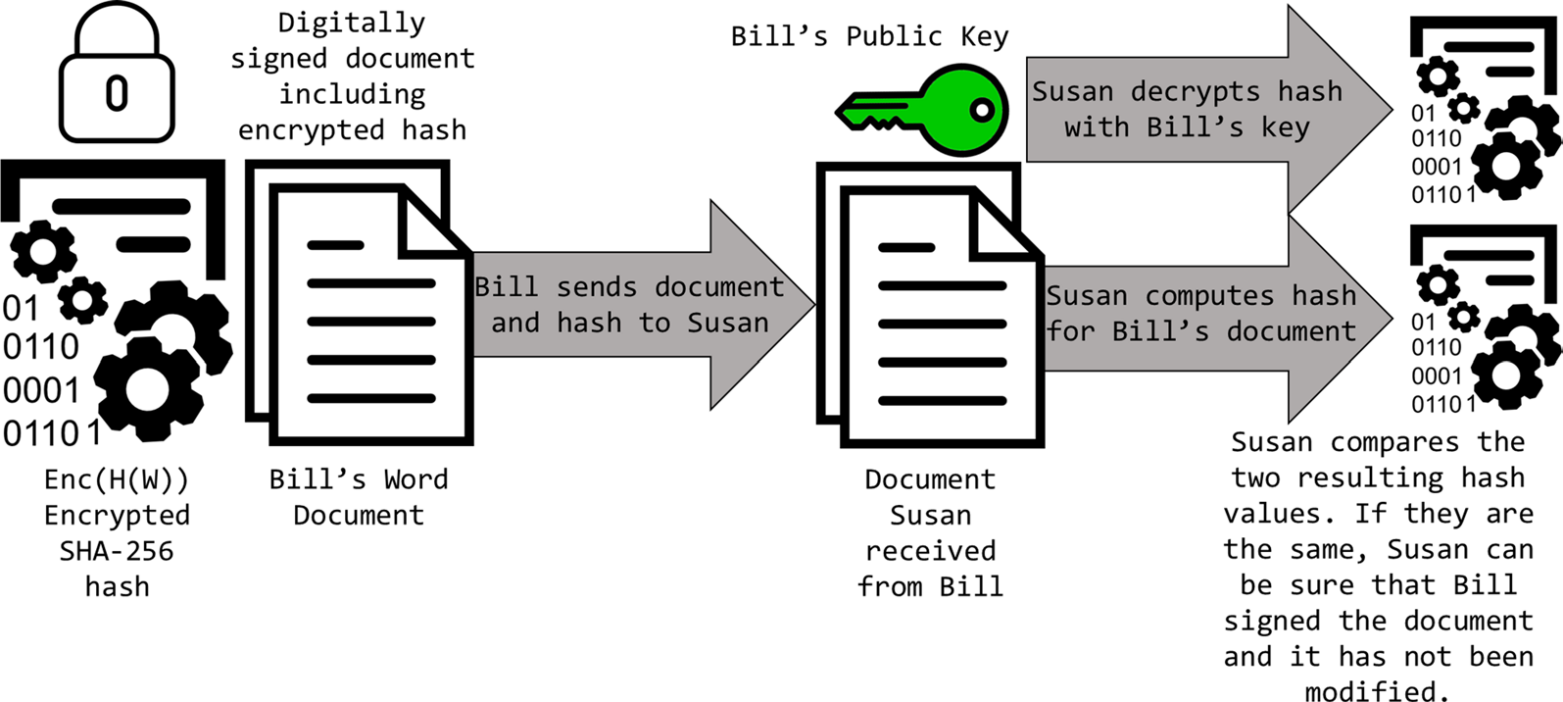
Verification of signing party upon receipt of encrypted document with hash value

Hashing and asymmetric encryption are excellent tools used in many different applications, and now that we know how they work, we will explore how they are implemented in blockchain technology. Recall two major roles that cryptographic functions hold: proof of data/asset ownership and data validation. The two cryptographic functions that we have discussed can be combined in this case. Imagine Bill has a word document containing sensitive information that he eventually wants to send to Susan; one technique to prove ownership is by first making a one-way hash of the document and then encrypting that hash. Encryption of the actual document can also be completed if warranted. The hash can only be decrypted through possession of the correct key, and then the unencrypted hash can be compared to a generated hash of the received document [5] (see Figs. 3, 4).

The final property of blockchain technology is immutability. Immutability implies some data, in this case a record of some type of transaction, cannot be tampered with or changed, only appended. Immutability is conferred from both the distributed nature and the cryptographic tools used for the blockchain. Notably, blockchains do not always have perfect immutability. Rather, through correct implementation and decentralisation, ensuring no party owns or controls the majority of the nodes in the blockchain network, is immutability able to be relied upon. Immutability is the by-product of cryptographic security and decentralisation. When considering immutability, one must be sure to recognise how it is generated from cryptography and decentralisation.

**Fig. 5:**
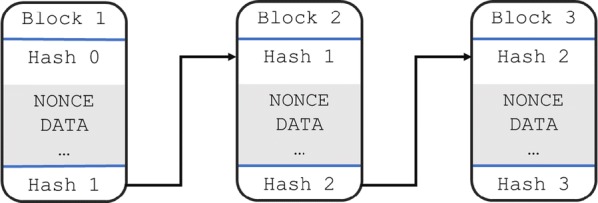
Simplified blockchain of three blocks

To understand how immutability confers security, we first need to examine a simplified anatomy of a block in the blockchain. A block is basically a container for some data spread across several nodes. In PoW, transaction fees are paid to miners to keep these nodes open, which in turn keeps the blockchain secure [1, 3]. Each block is numbered and possesses a hash and nonce value [1] (Fig. 5). The hash value links each block to the next, and the nonce is a variable value that ensures the correct hash is achieved in a PoW system (i.e., this is the value that miners are trying to find).

**Fig. 6:**
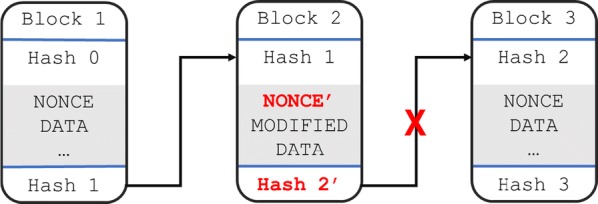
Simplified blockchain with adulterated block

The hash is one layer of protection leading to immutability. Since each block is linked to the next based on its hash, we know that any change that occurs in the data will drastically change the hash value [6]. Every block in the chain that comes after the adulterated block will be invalid (Fig. 6). This means that in order to change one block and re-mine its value to validate it, all blocks coming after will also need to be re-mined. This is a very high cost barrier to overcome for robust networks [7, 8]. Suppose that an attack here was successful though; our next layer of protection leading to immutability is the distribution and decentralisation. Not only does the entirety of the blockchain after the affected block need to be re-mined, but at least 51% of all distributed copies also need to be modified and subsequently re-mined for the change to take effect [1, 3]. This raises the cost of an attack even more, and demonstrates why, if implemented and maintained correctly, immutability is very reliable.

A glossary of blockchain and distributed ledger technology terms is presented in Table 1.

**Table 1:** Glossary of blockchain and distributed ledger technology terms [1, 3, 9]

Bitcoin	Cryptocurrency created by the person(s) named Satoshi Nakamoto in 2009. Introduced proof of work consensus for addressing the potential issue of double-spending of digital currency without a centralised form of authentication
Blockchain	A form of DLT where blocks of data are added sequentially and linked together with representative hash values
Ciphertext	Information (text) that has been encrypted (made unreadable) using an algorithm known as a cipher. This information can only be used if the appropriate cipher key is possessed
Consensus Algorithms	An algorithm or protocol used to find consensus, or agreement, among multiple distributed nodes. Consensus allows nodes to agree on updates to the blockchain itself. Examples include Proof of Work and Proof of Stake
DApps	Decentralised Applications (DApps) are applications written on the Ethereum blockchain with similar properties to a blockchain. They run on a decentralised network and remove the need for trust in any one agency. Contributions in computation to keep a DApp running pay out in a similar manner to contributions to blockchain nodes
Distributed Ledger Technology (DLT)	A database shared through consensus and spread among multiple sites, or nodes, and lacking centralised data storage
Ethereum	A blockchain alternative to the Bitcoin blockchain that introduces Smart Contracts, or scripting, and decentralised applications (DApps) by building in a Turing-complete programming language on top of the Ethereum blockchain
Fork	A split in the blockchain that could be caused by consensus protocol change (difference of opinion within community) or mining a different version of an existing block (attack) as examples. Forks can lead to small branches on the blockchain that are quickly abandoned or to new blockchains with their own supporters (Ethereum and Ethereum Classic)
Node	A device participating in the blockchain network. A blockchain network is comprised of distributed nodes each with their own copy of the blockchain’s information
Nonce	A random value used once to ensure the correct hash value is set during blockchain mining. This value is being mined to satisfy Proof of Work consensus
Smart Contracts	Programs or scripts written on the Ethereum blockchain that execute if a given set of specific requirements are met and that require no governing body to ensure their “payouts” are met properly

## Overview of blockchain in healthcare

With a better understanding of the fundamentals of blockchain technology, we will now examine some of the current state-of-the-art uses of blockchain in healthcare, as well as some proof of concepts (PoCs).

Blockchain technology and cryptocurrencies are being touted as the “solution” to problems in many different, disparate sectors throughout multiple industries. Public perception of this technology seems to be largely divided, with one group praising its abilities and implementation and another claiming that it is all hype and empty promises [10]. When viewing blockchain technology in light of the Gartner Hype Cycle [11, 12], it likely resides (as of mid-2018) between the ‘peak of inflated expectations’ and the ‘trough of disillusionment’ depending on perspective.

In the healthcare sector, “the core tenets of blockchain technology—a decentralised and encrypted way of distributing, sharing, and storing information—seem appealing for health data…. Yet blockchain technology raises its own security and privacy concerns just as it offers a new paradigm for distributing information” [10]. Blockchain technology also has the ability to act on clinical data sharing, either through storing the data itself or instructions on who can access that data (potentially through smart contracts), securing patient and provider identities and credentials, optimising management of the health supply chain, data sharing and consent for research and clinical trials (including data monetisation), and insurance and claims processing and detection/reduction of fraudulent activities.

As with any emerging technology in healthcare, the benefits of blockchain implementation are accompanied by its own set of challenges. Difficulties arise due to maintaining truly distributed patient data, the overwhelming amount of generated clinical data, and changes in consensus causing blockchains to fork. Many blockchain applications for storing patient data actually take a hybrid approach and store rules and references to data stored in a protected, centrally owned system or by utilising a private blockchain [13, 14]. This can appear to defeat the purpose of distribution altogether, as it is only one-step away from centralised ownership; however, implementation is key.

### Securing patient information and provider identities

Securing patient data for storage, patient access, and health system interoperability is a challenge for blockchain implementation due to its largely open nature. As said earlier, one solution to this is using a hybrid approach, but the issue of interoperability is still present using these models [14]. OmniPHR is a model focused on personal health record (PHR) distribution and interoperability [15]. The OmniPHR model stores PHR in encrypted datablocks that are distributed across nodes in their network. Each block is signed by the entity inserting the information into the datablock, which could be a healthcare professional, the patient, a caretaker, or a medical device [15]. Security is still a challenge—especially around ensuring only “authentic informants” have access to PHR data—but is a first step to completely decentralising patient information.

Securing provider identities and credentials is another area of focus. Piper Jaffray, a US investment bank and asset-managing firm, noted in a 2018 research report they published on blockchain in healthcare (28 pages; available by purchase from [16]), that data including education, licenses, and other credentials can be stored and updated in an immutable, verifiable way. The state of Illinois launched a blockchain initiative and partnered with Hashed Health [17], a blockchain healthcare company, to “explore opportunities to improve the efficiency and accuracy of the medical credentialing process in Illinois” [18]. By utilising a blockchain-based ledger to store medical credentials and licensures, sharing and verification of these licensures will become more efficient. The ledger can be viewed as the sole source of truth for existing credentials, allowing multiple parties to interact with this data in a much more streamlined manor [18]. Additional efforts for healthcare providers’ degree and credentialing have emerged including those by companies (e.g., Professional Credentials Exchange [19, 20]), educational institutions (e.g., Lipscomb University College of Pharmacy and Health Sciences [21]), and consortia collaborating on Decentralised Identity Hubs [22, 23].

### Health supply chain management

Supply chain management is necessary in any industry moving materials and goods in any way; however, pharmaceutical supply chain management is especially important to track the materials sourced for manufacturing, the manufacturing process itself, and distribution of the manufactured goods. Delivering substandard or counterfeit medications can have incredibly adverse effects on the people the medications were meant to help. In 2016, “the global market for fake, substandard, counterfeit, and grey market medicines [accounted] for up to $200 billion per year” [24]. Ensuring medication authenticity is vital for patient health and outcomes.

Substandard, falsified, and counterfeit medications are often seen in developing countries, or those with low-income markets. The amount of medication importation also plays a role in the verifiable authenticity of the product, especially with a weak or nonexistent supply chain management system. However, the United States has also been on the receiving end of fraudulent medications. In response to the threat of obtaining more fake medications, the US has started to implement the Drug Supply Chain Security Act (DSCSA). Key requirements for supply chain management technologies compliant to DSCSA are product identification, product tracing, product verification, detection and response to non-standard medications, notification upon identifying a non-standard medication, and the ability to store relevant information including licensures, verification, and product information [24]. Blockchain technology is applicable and compatible with each key requirement of DSCSA.

While pharmaceutical supply chain management and integrity are incredibly important, safety and security of medical devices and supplies can also be improved through blockchain implementation. Devices including implanted cardiac pacemakers and medication pumps can be compromised and controlled. Blockchain technology can be implemented in this field by holding unique device identifiers for each medical device (a requirement by the US FDA (Food and Drug Administration) and the EU) and by keeping track and issuing firmware updates by utilising smart contracts. A partnership between Edinburgh Napier University, NHS (National Health Service) Scotland, and Spiritus Development is leading an effort to use blockchain technology to track medical devices through their lifecycle [24, 25]. This device tracking has the potential to improve safety and efficiency of medical devices through more responsive device recalls and issued notices [24, 25]. Blockchain-based medical device tracking also can utilise immutability to prevent device loss, theft, or any other sort of malicious tampering.

Blockchain technology can improve supply chain management in a number of ways including: “… reducing or eliminating fraud and errors, reducing delays from paperwork, improving inventory management, identifying issues more rapidly, minimising courier costs, and increasing consumer and partner trust” [24, 26].

### Clinical research and data monetisation: giving patients the choice to share

A major benefit of blockchain technology is moving data ownership from institutions and corporations into the hands of the people who generated said data. This gives them control over who can see or interact with their data in any way. Not only does blockchain protect their data ownership, it also makes it easier to share data in a secure way while receiving benefits or payouts [27]. Health data can be used for clinical trial recruiting, can be monetised for research purposes, and shared with other healthcare professionals and EHRs (Electronic Health records) as needed for appropriate levels of care [28–30]. MedRec is an EHR implementation project started by the MIT (Massachusetts Institute of Technology) Media Lab and Beth Israel Deaconess Medical Center that takes a “decentralised approach to manage permissions, authorisation, and data sharing between healthcare systems” [13, 25].

Professor Andrew Lippman, associate director of the MIT Media Lab, recently spoke about MedRec at MIT Technology Review Conference. As he explained, full nodes act as the MedRec data server and maintain the blockchain. These nodes are themselves maintained by the entities generating data (medical professionals and institutions). Smart contracts define access and rights to data and is the “language” upon which the blockchain is defined. Patient wallets are how individuals interface with the blockchain. The wallets contain keys that provide access to the appropriate data [13, 14, 25]. MedRec does not put any actual health data onto the blockchain; Health data stays with the organisation that generated the data. This institution or organisation now acts as a data holder or repository when running the full node. When running the node, the organisation agrees to (1) be the repository of the smart contracts stored on the blockchain and the generated data, and (2) that they will obey instructions in the smart contracts to make the data available where needed and permissioned [13, 14, 25].

The MedRec blockchain sits somewhere in between the Bitcoin blockchain and a tradition database. In the Bitcoin blockchain, anyone can join and take part, which greatly increases complexity and expense to keep the chain running. MedRec restricts who can join the blockchain to medical providers and organisations. They run the full nodes, they maintain the data, and they keep the blockchain secure in a more efficient way than the Bitcoin blockchain could. The MedRec blockchain used to be maintained by medical researchers. As payment for maintaining the blockchain, they would gain access to random, anonymised health data for epidemiological research purposes. At the time of writing, MedRec has moved further to a proof of stake model. There are no transaction fees to move data around or use contracts. There is no coin that needs to be mined for transactions. It is maintained by the group of stakeholders made up by the healthcare organisations that take part in the MedRec blockchain.

### Claims processing and fraud detection

Claims processing has been identified as a target for blockchain disruption or enhancement, inclusive of streamlining preauthorisation submissions, health insurance claims adjudication, and eligibility management [30, 31]. One blockchain framework has explored doing so via a ‘decentralised infrastructure for healthcare service marketplaces’ using non-fungible tokens which would enable participants to negotiate and discover value [32]. Claims processing and related components tied to abbreviating payment cycles are also particularly fertile areas for integration of smart contract functionality to automate and accelerate. Recent legislative decisions in some regions are allowing enforcement of DLT smart contracts through their classification as legally binding [20, 33]. However, concerns have been expressed that what is evolving as a “patchwork” legislative approach to regulating these aspects of blockchain and DLT could complicate rather than clarify, especially if lawmakers and their advisors do not fully understand the scope of these emerging technologies [34]. Alternately, it is hoped that some of the same underlying features of blockchain that solved the “double spend” problem [1] along with the immutability of some ledgers will similarly help address the medical fraud, corruption, and abuse that is rampant in some health care systems [35, 36].

### Other emerging uses of blockchain in healthcare

In addition to the above-mentioned four major categories of blockchain use cases for healthcare, new categories are coalescing and individual use cases continue to emerge (see also the section below entitled ‘Geospatial blockchain use cases for smart healthy cities and regions’). These include, but are not limited to, public health surveillance [37], enhancing compliance in human subject regulations for IRBs (Institutional Review Boards) [29], improving medical records management [30], and leveraging genomic data in a broader way [38, 39]. Medication prescribing is another potential healthcare use case that could illustrate benefit from the transparency and share-ability of blockchains. A blockchain for prescriptions could be used as a ‘shared source of truth’, combating incorrect, outdated, and siloed data [10]. A blockchain for management of prescription data might also have the potential to enable new ways to interact with patients and their prescriptions, including writing a valid prescription to the blockchain without needing to specify a pharmacy and to allow partial filling of prescriptions across multiple pharmacies [10].

## Geospatial blockchain use cases for smart healthy cities and regions

The Internet of Things (IoT) is the foundation of the smart healthy cities and regions of today and tomorrow [40, 41]. To perform its ‘magic’ in improving citizens’ wellness and quality of life, the IoT generates and consumes big, versatile (and often geo-tagged) amounts of data. These data and their processing can greatly benefit from blockchain and related technologies. Ellehauge [42] cites the example of Uber, where a centralised approach with a ‘middleman’ owning and controlling data (and charging significant fees for matching consumers and service providers) can be replaced by a blockchain-style, distributed peer-to-peer alternative that offers users full control of their data whilst being cheaper to both clients and service providers.

Ellehauge [42] also explains the benefits of using blockchain technology to provide ‘truly public open data’ (but suitable business models are needed to cover the costs involved). Many current open data offerings are centralised, such as the UK Ordnance Survey map data (OS Maps), which, although free to end users, is financed through the taxpayer. IoT apps often rely on third parties for their geospatial elements, e.g., OS Maps or Google Maps data. But with access to truly publicly-distributed blockchain-style data, these apps can become more reliable and cheaper to run and sustain. With blockchain-style open data, no one can restrict access to the data (unlike with a centralised system), and costs can be kept to a minimum, thanks to the open nature of competing nodes and contributors. Geospatial data contributors can be rewarded with some form of tokens, and a public record can be kept of all changes and contributions made.

The market for IoT devices and apps that negotiate with, and pay, each other for secure, safe operation and services, e.g., mobile and wearable devices that pay for public transportation [43], and autonomous connected devices and vehicles for smart city emergency/disaster response, such as a drone defibrillator, or a drone for the delivery of ordered medicines and medical supplies [44], or a self-driving ambulance car (or helicopter), is expected to grow in the near future. Distributed peer-to-peer apps powering these smart drones and vehicles would cut out the ‘middleman’ and the dependence on third-party providers for navigation and other geospatial data [42, 45]. Dasgupta [46] mentions how a well-conceived blockchain can mitigate the possibility of an IoT-powered autonomous vehicle being hijacked and driven to a wrong location. If we consider the data carrying the instructions to the vehicle as transactions, and the network is on a blockchain, then the process of consensus would help validate these transactions, trapping any illegal ones, and weeding out the wrong instructions they carry.

Citizen engagement in the crowdsourcing of geo-tagged data can be combined with augmented reality (AR) and blockchain technology (blockchain-enabled AR) in powerful new crisis mapping and recovery scenarios, e.g., in the production and real-time updating of an augmented crisis map for navigating a disaster-stricken area, in which geo-tagged AR objects providing critical contextual information and advice are superimposed onto the real world scene on user’s smartphone (such as a ‘Do Not Drive; Cable Wires Ahead’ message when approaching a flooded zone). The crowdsourced data objects can be blockchain-validated, credited and rewarded [47].

Implementation-wise, FOAM [48] is a good example of a geospatially-enabled blockchain using a crypto-spatial coordinate (CSC) system. A FOAM blockchain does not just record an entry’s specific time, but also requires and validates its associated proof of location, giving an immutable spatial context that regular blockchains lack, and allowing the accurate mapping of physical world events in a temporal sequence [46, 49, 50].

### Challenges

Among the challenges facing geospatial blockchain implementations today, there are three particularly pressing ones (besides the above-mentioned need for sustainable business models) upon which the future success and mainstream adoption of the technology will be hinging. These three challenges require careful consideration and innovative solutions (both technical and regulatory) to address them. The first issue is interoperability, to have blockchains from different providers and services seamlessly talk to each other as appropriate [51]. The second issue is blockchain security [52]. After all, the whole rationale of using a blockchain is to let people who did not previously know or trust one another share data in a secure, tamperproof way. But the security of even the best-conceived blockchain can fail in some scenarios (e.g., the so-called ‘51% attacks’) [52, 53], calling for adequate pre-emptive mechanisms to be put in place in order to mitigate or prevent blockchain security breaches. The third challenge is to adequately reconcile blockchain’s promise of transparency with the European Union’s now much stricter privacy rules under GDPR (General Data Protection Regulation) that require personal data to be deletable on demand [54].

## Conclusions

At the time of writing, a PubMed query using the keyword ‘blockchain’ retrieved 40 indexed papers [55], a reflection of the growing interest in blockchain amongst the medical and healthcare research and practice communities. Blockchain technologies are being investigated for use in public health and healthcare in numerous disruptive ways. Their foundations of decentralisation, cryptographic security and immutability make blockchain a strong contender in reshaping the healthcare landscape of the world abroad.

Blockchain solutions are currently being explored for:

1. securing patient and provider identities;
2. managing pharmaceutical and medical device supply chains;
3. clinical research and data monetisation, e.g., [56–58];
4. medical fraud detection;
5. public health surveillance, e.g., by the US CDC (Centers for Disease Control and Prevention) for sharing public health data to help public health workers respond faster to a crisis [59];
6. enabling truly public and open geo-tagged data;
7. powering many IoT-connected autonomous devices, wearables, drones and vehicles, via the distributed peer-to-peer apps they run, to deliver the full vision of smart healthy cities and regions; and
8. blockchain-enabled augmented reality in crisis mapping and recovery scenarios, including mechanisms for validating, crediting and rewarding crowdsourced geo-tagged data, among other emerging blockchain use cases.

Geospatially-enabled blockchain solutions exist today that use a crypto-spatial coordinate system to add an immutable spatial context that regular blockchains lack. These geospatial blockchains do not just record an entry’s specific time, but also require and validate its associated proof of location, thus facilitating the accurate spatiotemporal mapping of physical world events.

Blockchain and DLT have the potential to benefit all the above application areas and many more, but also face similar challenges as those faced by any other technology threatening to disintermediate legacy processes and commercial interests, namely the challenges of blockchain interoperability, security and privacy, as well as the need to find suitable and sustainable business models of implementation. Nevertheless, we expect blockchain technologies to get increasingly powerful and robust, as they become coupled with artificial intelligence (AI) [60] in various real-word healthcare solutions. AI-mediated health data exchange on blockchains will play important roles in shaping the future of these technologies in healthcare [61].
